# QSAR Modeling and Molecular Docking Analysis of Some Active Compounds against* Mycobacterium tuberculosis* Receptor (Mtb CYP121)

**DOI:** 10.1155/2018/1018694

**Published:** 2018-05-10

**Authors:** Shola Elijah Adeniji, Sani Uba, Adamu Uzairu

**Affiliations:** Department of Chemistry, Ahmadu Bello University, Zaria, Nigeria

## Abstract

A quantitative structure-activity relationship (QSAR) study was performed to develop a model that relates the structures of 50 compounds to their activities against* M. tuberculosis*. The compounds were optimized by employing density functional theory (DFT) with B3LYP/6-31G^⁎^. The Genetic Function Algorithm (GFA) was used to select the descriptors and to generate the correlation model that relates the structural features of the compounds to their biological activities. The optimum model has squared correlation coefficient (*R*^2^) of 0.9202, adjusted squared correlation coefficient (*R*_adj_) of 0.91012, and leave-one-out (LOO) cross-validation coefficient (*Q*_cv_^2^) value of 0.8954. The external validation test used for confirming the predictive power of the built model has *R*^2^pred value of 0.8842. These parameters confirm the stability and robustness of the model. Docking analysis showed the best compound with high docking affinity of −14.6 kcal/mol which formed hydrophobic interaction and hydrogen bond with amino acid residues of* M. tuberculosis* cytochromes (Mtb CYP121). QSAR and molecular docking studies provide valuable approach for pharmaceutical and medicinal chemists to design and synthesize new anti-*Mycobacterium tuberculosis* compounds.

## 1. Introduction

*Mycobacterium tuberculosis* is a bacterial species responsible for causing tuberculosis (TB). It mainly affects the lungs and other parts of the body such as spine, kidney, and brain unless urgent treatment is provided. Tuberculosis remains one of the most prevalent infectious bacterial diseases, resulting in the death of 1.4 million people worldwide [1]. There are drugs like isoniazid, rifampicin, ciprofloxacin, and ethambutol available as a cure for tuberculosis.

However, due to the emergence of multidrug-resistant (MDR) and extensively drug-resistant (XDR) tuberculosis, this poses a big challenge towards the successful treatment of tuberculosis [2]. This led to development of new therapeutics against diverse strains of* M. tuberculosis* [3]. New synthesized 1,2,4-Triazole derivative compounds demonstrate tuberculosis inhibition activity [4]. Synthesis of novel molecules is typically developed using a trial-and-error approach, which is time-consuming and costly.

Quantitative structure-activity relationship (QSAR) plays a crucial part in novel drug design via a ligand-based approach [5]. The key success of the QSAR method is the possibility to predict the properties of new chemical compounds without the need to synthesize and test them. This technique is broadly utilized for the prediction of physicochemical properties in the chemical, industrial, pharmaceutical, biological, and environmental spheres [6]. Moreover, the QSAR strategies save resources and accelerate the process of developing new molecules for use as drugs, materials, and additives or for whatever purposes [7]. Meanwhile molecular docking is a computational method used to determine the binding strength between the active site residues and specific molecule(s) [8]. Molecular docking is expedient tool used in the drug discovery field to investigate the binding compatibility of molecules (ligands) to target (receptor) [9].

The aim of this research was to develop QSAR model to predict the activity of 1,2,4-Triazole derivatives as potent anti-*Mycobacterium tuberculosis* compounds and to elucidate the interaction between the inhibitor molecules and* Mycobacterium tuberculosis* target site.

## 2. Materials and Methods

### 2.1. Data Collection

Fifty molecules of 1,2,4-Triazole derivatives as potent antitubercular agents that were used in this study were obtained from the literature [4].

### 2.2. Biological Activities (BA)

The biological activities of 1,2,4-Triazole derivatives against* Mycobacterium tuberculosis* in aerobic active stage were initially expressed in percentage (%) and then converted to logarithm unit using (1) in order to increase the linearity and approach normal distribution of the activity values. The observed structures and the biological activities of these compounds were presented in Table 1.

(1) pBA=log⁡molecular  weightg/molDoseg/mol·percentage%100−percentage%.

### 2.3. Geometry Optimization

The chemical structures of the molecules were drawn with ChemDraw Ultra Version 12.0. Each molecule was first preoptimized with the molecular mechanics (MMFF) and further reoptimized with density functional theory (DFT) utilizing the B3LYP and 6-31G^*∗*^ basis set [10, 11] with the aid of Spartan 14 Version 1.1.0 software.

### 2.4. Molecular Descriptor Calculation

Molecular descriptors are mathematical values that describe the properties of a molecule. Descriptors calculation for all the 50 molecules of 1,2,4-Triazole derivatives was done using PaDEL-Descriptor Version 2.20 software. A total of 1876 molecular descriptors were calculated.

### 2.5. Normalization and Data Pretreatment

The descriptors' values were normalized using (2) in order to give each variable the same opportunity at the onset to influence the model [12].

(2) $$\begin{array}{c} X=\frac{X_{1}-X_{min}}{X_{max}-X_{min}}, \end{array}$$

where *X*_*i*_ is the value of each descriptor for a given molecule and *X*_max_ and *X*_min_ are the maximum and minimum value for each column of descriptors *X*. The normalized data were subjected to pretreatment using data pretreatment software in order to remove noise and redundant data.

### 2.6. Training and Test Set

The dataset was split into training set and test set by employing Kennard and Stone's algorithm. The training set comprises 70% of the dataset which was used to build the model, while the remaining 30% of the dataset (test set) was used to validate the built model.

### 2.7. Relative Importance of Each Descriptor in the Model

Absolute value of the mean effect of each descriptor was used to evaluate the relative importance and contribution of the descriptor to the model. The mean effect is defined as

(3) $$\begin{array}{c} ME=\frac{\beta_{j}\sum_{i}^{n}D_{j}}{\sum_{j}^{m}\left(\beta_{j}\sum_{i}^{n}D_{j}\right)}, \end{array}$$

where ME is the mean effect of a descriptor* j* in a model, *β*_*j*_ is the coefficient of the descriptor *j* in that model, *D*_*j*_ is the value of each descriptor in the data matrix for each molecule in the training set, *m* is the number of descriptors that appear in the model, and *n* is the number of molecules in the training set [13].

### 2.8. Degree of Contribution of Selected Descriptors

Contribution of each descriptor in the model was measured by calculating their standardized regression coefficients (*b*_*j*_^*s*^) using (6).

(4) $$\begin{array}{c} b_{j}^{s}=\frac{S_{j}b_{j}}{S_{y}}, \end{array}$$

where *b*_*j*_ is the regression coefficient of descriptor *j*. *S*_*j*_ and *S*_*y*_ are the standard deviations for each descriptor and activity, respectively. Statistical property *b*_*j*_^*s*^ allows one to assign a greater importance to those molecular descriptors that exhibit larger absolute standardized coefficients

### 2.9. Internal Validation of Model

Internal validation of the model was carried out using Materials Studio Version 8 software by employing the Genetic Function Approximation (GFA) method. The models were estimated using the LOF. The LOF is measured using a slight variation of the original Friedman formula, so that the best fitness score can be received. LOF is expressed as follows [14]:

(5) $$\begin{array}{c} LOF=\frac{SEE}{{\left(1-\left(C+d\times p\right)/M\right)}^{2}}, \end{array}$$

where SSE is the sum of squares of errors, *C* is the number of terms in the model, *d* is a user-defined smoothing parameter, *p* is the total number of descriptors contained in the model, and *M* is the amount of data in the training set. SEE is defined as [15]

(6) $$\begin{array}{c} SEE=\sqrt{\frac{{\left(Y_{exp}-Y_{pred}\right)}^{2}}{N-P-1}}. \end{array}$$

The square of the correlation coefficient (*R*^2^) defines the fraction of the total variation attributed to the model. The closer the value of *R*^2^ to 1.0, the better the model generated. *R*^2^ is expressed as

(7) $$\begin{array}{c} R^{2}=1-\left[\;\frac{\sum {\left(Y_{exp-Y_{pred}}\right)}^{2}}{\sum {\left(Y_{exp-{\bar{Y}}_{training}}\right)}^{2}}\;\right], \end{array}$$

where *Y*_exp_, *Y*_pred_, and ${\bar{Y}}_{training}$ are the experimental activity, the predicted activity, and the mean experimental activity of the samples in the training set, respectively.

Value of *R*^2^ varies directly with the increase in number of descriptors. Thus, *R*^2^ is not reliable to measure the goodness of fit of the model. Therefore, *R*^2^ is adjusted for the number of explanatory variables in the model. The adjusted *R*^2^ is defined as

(8) $$\begin{array}{c} {R^{2}}_{adj}=\frac{R^{2}-k\left(n-1\right)}{n-p+1}, \end{array}$$

where *k* is the number of independent variables in the model and *n* is the number of descriptors.

The strength of the QSAR equation to predict bioactivity of a compound was determined using the leave-one-out cross--validation method. The revised formula for cross-validation regression coefficient (*Q*_cv_^2^) is

(9) $$\begin{array}{c} Q_{cv}^{2}=1-\left[\;\frac{\sum {\left(Y_{pred-Y_{exp}}\right)}^{2}}{\sum {\left(Y_{exp-{\bar{Y}}_{training}}\right)}^{2}}\;\right], \end{array}$$

where *Y*_pred_, *Y*_exp_, and ${\bar{Y}}_{training}$ are the predicted, experimental, and mean values of experimental activity of the training set.

### 2.10. External Validation of Model

External validation of model was assessed by *R*_test_^2^ value. The closer the value of *R*_test_^2^ to 1.0, the better the model generated.

(10) $$\begin{array}{c} R_{test}^{2}=1-\frac{\sum {\left(Y{pred}_{test}-Y_{{exp}_{test}}\right)}^{2}}{\sum {\left(Y{pred}_{test}-{\bar{Y}}_{training}\right)}^{2}}, \end{array}$$

where *Y*pred_test_ and *Y*_exp_test__ are the predicted and experimental activity test set, while ${\bar{Y}}_{training}$ represents mean values of experimental activity of the training set.

### 2.11. *Y*-Randomization Test

*Y*-Randomization test is another useful external validation parameter to confirm that the built QSAR model is strong and is not inferred by chance. The *Y*-Randomization test was performed on the training set data [16]. For the built model to pass the *Y*-Randomization test, *cR*_*p*_^2^ should be more than 0.5.

(11) $$\begin{array}{c} cR_{p}^{2}=R\times {\left[\;R^{2}-{\left(\;R_{r}\;\right)}^{2}\;\right]}^{2}, \end{array}$$

where *cR*_*p*_^2^ is coefficient of determination for *Y*-Randomization, *R* is coefficient of correlation for *Y*-Randomization, and *R*_*r*_ is average “*R*” of random models.

### 2.12. Evaluation of the Applicability Domain of the Model

Assessment of the applicability domain of a QSAR model is an important approach to confirm that the model built is able to make good predictions within the chemical space for which it was developed [16]. The leverage approach was employed to describe the applicability domain of the QSAR model [17]. Leverage of a given chemical compound is defined as follows:

(12) $$\begin{array}{c} hi=X_{i}{\left(\;X^{T}X\;\right)}^{-1}X_{i}^{T}, \end{array}$$

where *hi* is the leverage of each compound, *X*_*i*_ is the descriptor row-vector of the query compound *i*, and *X* is the *n* × *k* descriptor matrix of the training set compounds used to build the model. As a prediction tool, the warning leverage (*h*^*∗*^) is the limit of normal values for *X* outliers and is defined as

(13) $$\begin{array}{c} h^{*}=3\frac{\left(d+1\right)}{m}, \end{array}$$

where *m* is the number of training compounds and *d* is the number of descriptors in the model. The Williams plot, a plot of standardized residual versus leverage value, was employed to elucidate the relevance area of the model in terms of chemical space. Data is said to be an outlier if the standardized cross-validated residual produced by the model is greater than ±3.

### 2.13. Quality Assurance of the Model

The fitting ability, stability, robustness, reliability, and predictive ability of the developed models were evaluated by internal and external validation parameters. The validation parameters were compared with the minimum recommended value for a generally acceptable QSAR model [17] shown in Table 2.

### 2.14. Docking Studies

**Figure 1:**
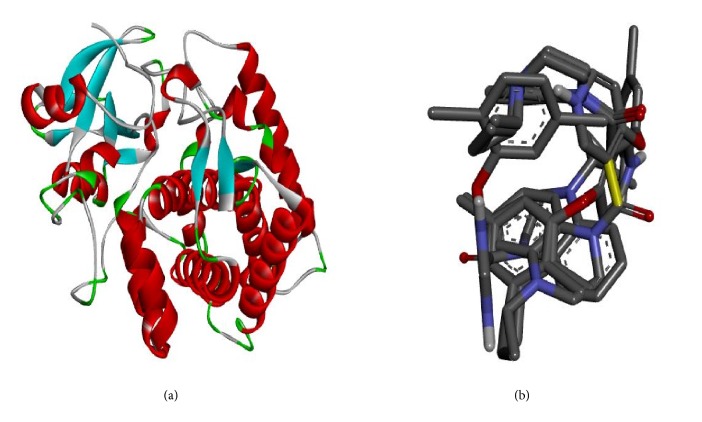
(a) Prepared structure of Mtb CYP121. (b) 3D structures of the prepared ligands.

Molecular docking study was carried out in order to elucidate which of the 1,2,4-Triazole derivatives has the best binding affinity against Mtb CYP121. The structure of Mtb CYP121 used in the study was obtained from protein data bank with PDB code 51BG. The prepared ligand and receptor were shown in Figure 1. The optimized structures of 1,2,4-Triazole derivatives initially saved as SDF files were converted to PDB files using Spartan 14 V 1.1.4. The prepared ligands were docked with prepared structures of Mtb CYP121 using AutoDock Vina incorporated in PyRx software. The docked results were compiled, visualized, and analyzed using Discovery Studio Visualizer.

## 3. Results and Discussion

QSAR was performed to investigate the structure-activity relationship of 50 compounds as potent antitubercular agents. The nature of model in a QSAR study is expressed by its fitting ability, stability, robustness, reliability, and forecast capacity.

Experimental and predicted activities for 1,2,4-Triazole derivatives were presented in Table 3. The low residual value between experimental and predicted activity indicates that the model is of high predictability.

The genetic algorithm-multiple linear regression (GA-MLR) investigation led to the selection of six descriptors that were used to assemble a linear model for calculating predictive activity on* Mycobacterium tuberculosis*. Five QSAR models were built using Genetic Function Algorithm (GFA), but due to the statistical significance, model 1 was selected and reported as given below:

(14) pBA=−0.307001458  AATS7s+1.528715398  nHBint3+3.976720227  minHCsatu+0.016199645  TDB9e+0.089381479  RDF90i−0.107407822  RDF110s+4.057082751.

 
*N*_train_ = 35, *R*^2^ = 0.92023900, *R*_adj_ = 0.91017400, *Q*_cv_^2^ = 0.89538600, and the external validation for the test set was found to be *R*^2^pred = 0.8842.

All the validation parameters for this mode were reported in Table 4 and were all in agreement with parameters presented in Table 2, which actually confirmed the robustness of the model.

The QSAR model generated in this research was compared with the models obtained in the literature [18, 19] as shown below:

(15) pMIC=4.77374+/−0.03903−0.18609+/−0.04924AATS4i+0.50382+/−0.05235SCH-3−0.44712+/−0.06573AVP-1−0.22376+/−0.05623maxHCsats−0.18403+/−0.04374PSA.

*N*_train_ = 16, *R*^2^ = 0.9184, *Q*_cv_^2^ = 0.84987, and *R*^2^pred = 0.79343 [18].
  (16) pIC50=−2.040810634∗nCl−19.024890361∗MATS2m+1.855704759∗RDF140s+6.739013671.
*N*_train_ = 27, *R*^2^ = 0.9480, *R*_adj_ = 0.9350, *Q*_cv_^2^ = 0.87994, and *R*^2^pred = 0.76907 [19].

From the above models, it could be seen that maxHCsats and 3D-radial distribution function (RDF) descriptors were also observed in the model generated in this research. This indicates that these descriptors have great influence on the activities of the inhibitory compounds against* Mycobacterium tuberculosis*. The validation parameters reported in this work and those reported in the literature were all in agreement with parameters presented in Table 2, which actually confirmed the robustness of the model.

Descriptive statistics of the activity values of the training and test set data reported in Table 5 show that test set value range (8.2854 to 4.9074) was within the training set value range (8.0899 to 4.7441). Also, the mean and standard deviation of the test set activity value (6.4989 and 0.93) were approximately similar to those of the training set value (6.6222 and 0.96). This indicates that the test set is interpolative within the training. Therefore, Kennard and Stone's algorithm employed in this study was able to generate a test set that is a good reflection of the training set.

The name and symbol of the descriptors used in the QSAR optimization model were reported in Table 6. The presence of the three 2D and three 3D descriptors in the model suggests that these types of descriptors are able to characterize better anti-*Mycobacterium tuberculosis* activities of the compounds. Pearson's correlation matrix and statistics of the six descriptors employed in the QSAR model were reported in Table 7, which shows clearly that the correlation coefficients between each pair of descriptors are very low; thus, it can be inferred that there exists no significant intercorrelation among the descriptors used in building the model. The absolute *t*-statistics value for each descriptor is greater than 2 at 95% significant level, which indicates that the selected descriptors were good. The estimated Variance Inflation Factor (VIF) values for all the descriptors were less than 4, which imply that the model generated was statistically significant and the descriptors were orthogonal.

The mean effect (ME) values and standard regression coefficient (*b*_*j*_^*s*^) reported in Table 8 provide important information on the effect of the molecular descriptors and the degree of contribution in the developed model. The signs and the magnitude of these descriptors combined with their mean effects indicate their individual strength and direction in influencing the activity of a compound. The null hypothesis says that there is no significant relationship between the descriptors and the activities of the inhibitor compounds. The *p* values of the descriptors at 95% confidence limit shown in Table 8 are all less than 0.05. This implies that the alternative hypothesis is accepted. Hence, there is a relationship between the descriptors used in generating the model and the activities of the inhibitor compounds which take preference over the null hypothesis.

*Y*-Randomization parameter test was reported in Table 9. The low *R*^2^ and *Q*^2^ values for several trials confirm that the developed QSAR model is robust, while the *cR*_*p*_^2^ value greater than 0.5 affirms that the created model is powerful and is not inferred by chance.

### 3.1. Interpretation of Selected Descriptors

AATS7s is average Moreau-Broto Autocorrelation-lag 7/weighted by I-state autocorrelation descriptor. It is based on spatial dependent autocorrelation function that measures the strength of the relationship between observations (atomic or molecular properties) and space separating them (lag). This descriptor is obtained by taking the molecule atoms as the set of discrete points in space and an atomic property as the function evaluated at those points. When this descriptor is calculated on molecular graph, the lag coincides with the topological distance between any pair of the vertices. AATS7s is defined on the molecular graphs using atomic masses (*m*), Sanderson electronegativity (*e*), and inductive effect of pairs of atoms 7 bonds apart as the weighting scheme. These observations suggested that atomic masses and electronic distribution of the atoms that made up the molecule had significant effect on the antitubercular activity of the dataset. In addition, the signs of the regression coefficients for each descriptor indicated the direction of influence of the descriptors in the models such that positive regression coefficient associated with a descriptor will augment the activity profile of a compound, while the negative coefficient will diminish the activity of the compound.

Electrotopological state atom-type descriptor nHBint3 represents count of E-state descriptors of strength for potential hydrogen bonds of path length 3. It is a spatial dependent 2D autocorrelation descriptor with the incorporation of Moran coefficient (index) in the measurement of the strength of the relationship between observations and space separating them. This Moran autocorrelation descriptor contained in the model reported in this study was defined on the molecular graphs using atomic masses (*m*), Sanderson electronegativity (*e*), and inductive effect of pairs of atom 3 bonds apart as the weighting scheme. These observations supported the claim that atomic masses and electronic distribution had significant effect on the antitubercular activities of the molecules The positive mean effect of this descriptor indicates that the inhibitory activity of 1,2,4-Triazole derivatives will increase with hydrogen bonds of path length 3.

minHCsatu (minimum atom-type H E-state: H on C sp3 bonded to unsaturated C) is a 2D electrotopological state (E-state indices) atom-type descriptor. In general, E-state indices encode the intrinsic electronic state of each atom as perturbed by the electronic influences of all other atoms in the molecule within the context of the topological character of the molecule. maxHCsatu favors the addition of –CH3 to unsaturated C atom, for example, in benzene ring. Positive contribution of minHCsatu indicates that the inhibitory activity of 1,2,4-Triazole derivatives will increase with increase in the molecular descriptor.

TDB9e (3D topological distance based autocorrelation-lag 9/weighted by Sanderson electronegativities) is positively correlated to the anticonvulsant activity, meaning that increase in its value augments the activity of the studied compounds. The descriptor measures the strength of the connection between atomic charges 9 bonds apart. The number of rings in the molecular system tends to increase the values of this descriptor as observed for molecules. This may be due to increase in the amount of *π*-electrons in the molecular system, bringing about increase in the charge difference between atoms 9 bonds apart. The positive mean effect indicates a positive impact on the activity of the inhibitory compounds, which means that increasing the value of this descriptor produces higher activity of these compounds.

RDF90i and RDF110s are 3D radial distribution functions at 2.5 and 7.0 interatomic distance weighted by atomic masses. The radial distribution function is probability distribution to find an atom in a spherical volume of radius. RDF descriptors are independent of the size and rotation of the entire molecule. They describe the steric hindrance or the structure-activity properties of a molecule. The RDF descriptor provides valuable information about the bond distances, ring types, planar and nonplanar systems, and atom types. The presence of these descriptors in the model suggested the occurrence of a linear relationship between antitubercular activity and the 3D molecular distribution of atomic masses in the molecules calculated at radius of 2.0 Å and 7.0 Å from the geometrical centers of each molecule. RDF90i with positive mean effect (MF) indicates positive impact on the activity, while RDF110s with negative mean effect (MF) indicates negative contribution on the activity.

**Figure 2:**
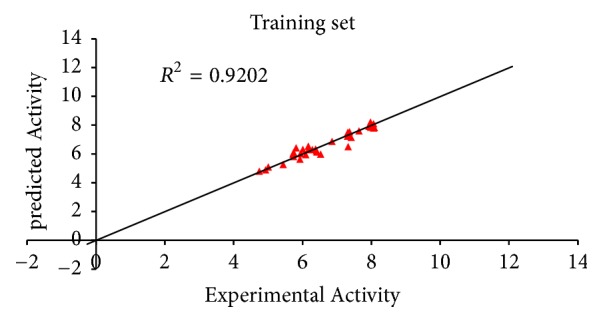
Plot of predicted activity against experimental activity of training set.

**Figure 3:**
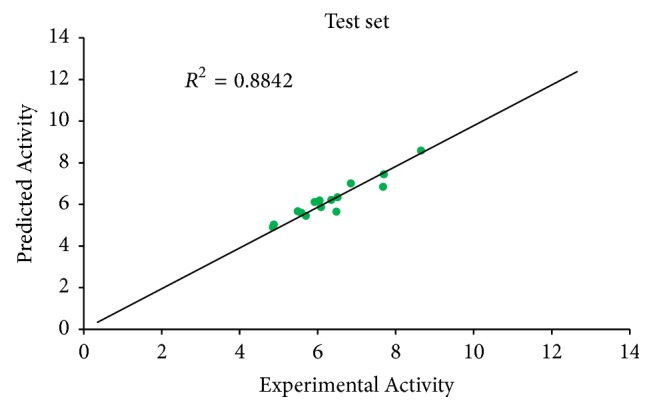
Plot of predicted activity against experimental activity of test set.

**Figure 4:**
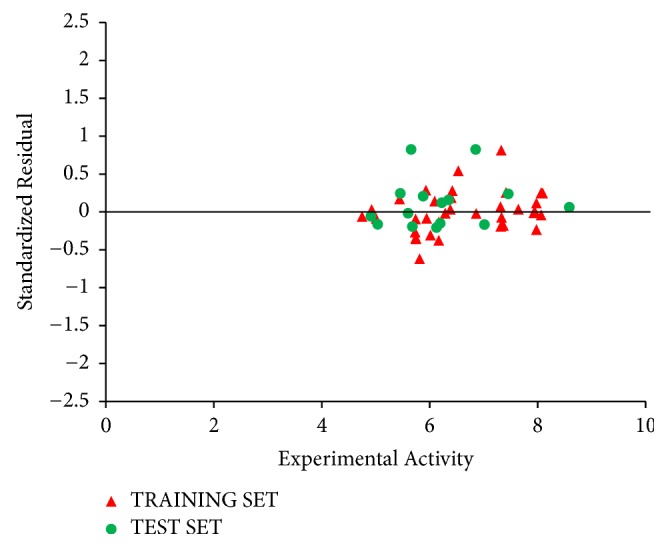
Plot of standardized residual activity versus experimental activity.

Predicted activity against experimental activity of training and test set was shown in Figures 2 and 3. The *R*^2^ value of 0.9202 for training set and *R*^2^ value of 0.8842 for test set recorded in this study were in agreement with GFA-derived *R*^2^ value reported in Table 2. This confirms the stability, reliability, and robustness of the model. Plot of standardized residual versus experimental activity shown in Figure 4 indicates a symmetric distribution or random scattering of data points above and below the standardized residual line equal to zero. Also, all the data points were within the boundary defined by standardized residual of ±2. Thus, it implies that there was no systemic error in model developed as the spread of residuals was pragmatic on both sides of zero [20].

**Figure 5:**
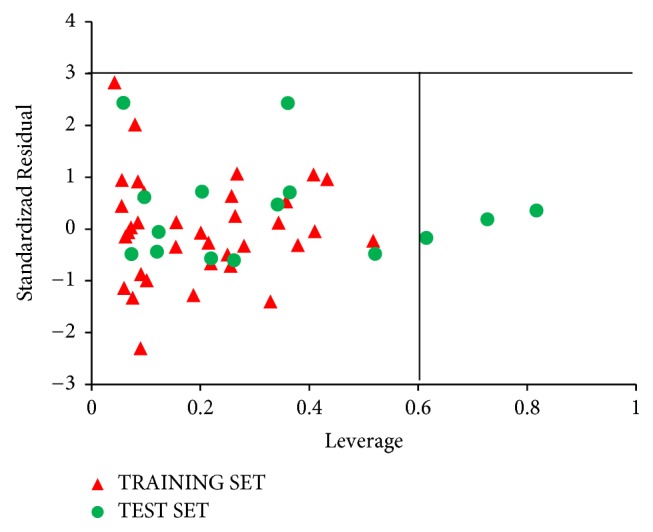
The Williams plot of the standardized residuals versus the leverage value.

The standardized residuals in the dataset were plotted against their leverages for every compound, leading to discovery of outliers and influential molecules in the models. The Williams plot of the standardized residuals versus the leverage value is shown in Figure 5. From our result, it is evident that no outlier is found, since all the compounds for both the training and test set were within the applicability domain of the square area except for three compounds that are structurally influential molecules (i.e., compounds 50, 39, and 36). These three compounds are said to be structurally influential molecules, since their leverage values are greater than the warning leverage (*h*^*∗*^ = 0.60). Their high leverage values are responsible for swaying the performance of the model. This was attributed to strong differences in their chemical structures compared to other compounds in the dataset.

### 3.2. Molecular Docking

Molecular docking study was carried out between the target (Mtb CYP121) and 1,2,4-Triazole derivatives. All the compounds were found to inhibit the receptor by occupying the active sites of the target protein (Mtb CYP121).

For target protein, binding affinity values for all the compounds range from −5.1 to −14.6 kcal/mol as reported in Table 10. However, four ligands (compounds 7, 8, 13, and 14) have higher binding score, which ranges from −10.0 to 14.6 kcal/mol, which were greater than their co-ligands.

**Figure 6:**
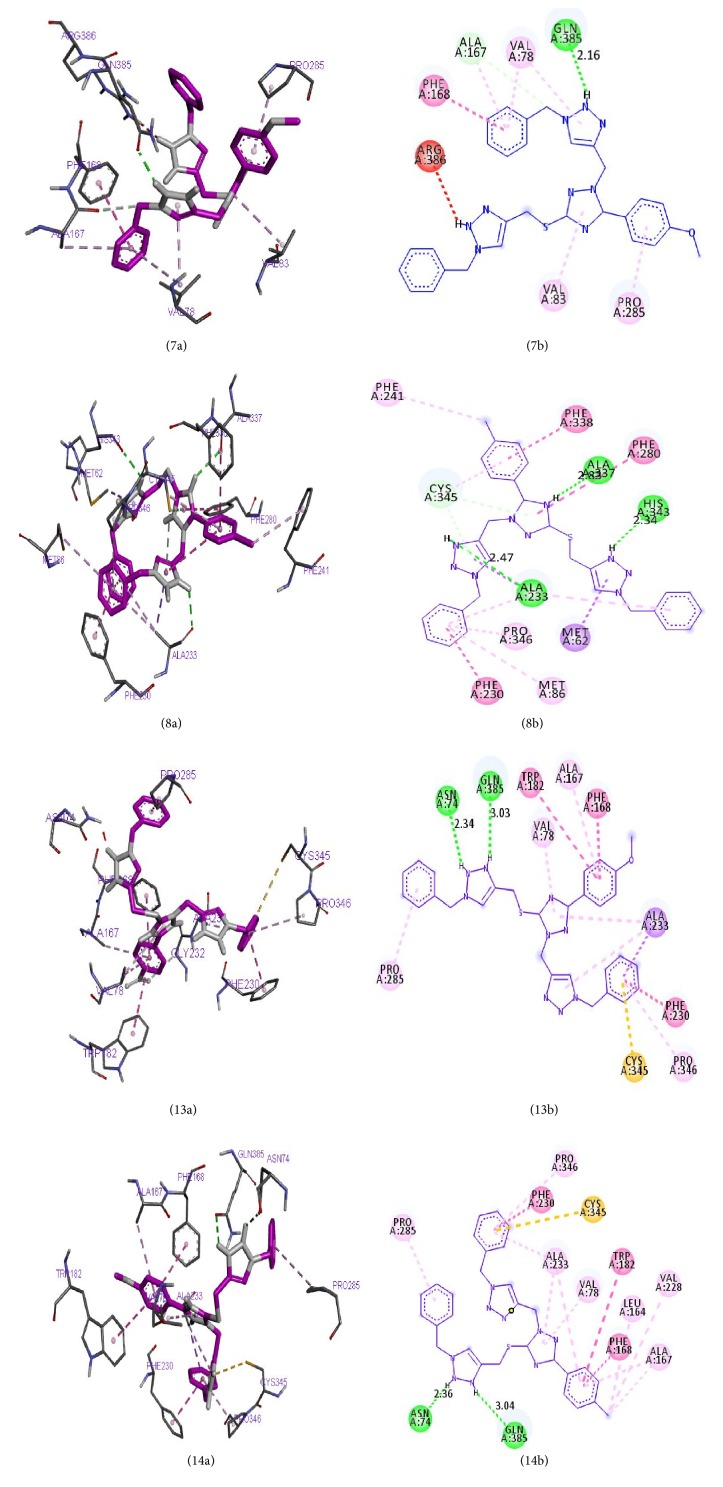
(7a) and (7b) show the 3D and 2D interactions between Mtb CYP121 and ligand 7. (8a) and (8b) show the 3D and 2D interactions between Mtb CYP121 and ligand 8. (13a) and (13b) show the 3D and 2D interactions between Mtb CYP121 and ligand 13. (14a) and (14b) show the 3D and 2D interactions between Mtb CYP121 and ligand 14.

These four ligands were visualized and analyzed in Discovery Studio Visualizer as shown in Figure 6. Binding affinity, hydrogen bond, and hydrophobic bond of ligands 7, 8, 13, and 14 with* M. tuberculosis* target (Mtb CYP121) are reported in Table 11. Ligand (compound 7) formed hydrophobic interactions with VAL83 PRO285, VAL78, and ALA167 of the target site. In addition, ligand 7 also forms hydrogen bonds (2.16131 Å) with GLN385. Ligand 8 made three hydrogen bonds (2.82894, 2.34089, and 2.47314 Å) with ALA337, HIS343, and ALA233 of the target, while hydrophobic interactions were observed with PHE280, ALA233, CYS345, MET86, ALA233, and PRO346. Ligand 13 made two hydrogen bonds (2.34218 and 3.0328 Å) with ASN74 and GLN385, while VAL78, ALA233, PRO285, ALA233, PRO346, and ALA167 form the hydrophobic interaction. Ligand 14 formed hydrophobic interaction with LEU164, VAL228, VAL78, ALA233, PRO285, ALA233, PRO346, ALA167, and ALA233, while two hydrogen bonds (2.36479 and 3.03627 Å) were formed between ASN74 and GLN385 of the target.

## 4. Conclusion

The model with 2D and 3D descriptors is of higher excellence and presents a satisfactory correlation with the anti-*Mycobacterium tuberculosis* activity. The combination of 2D and 3D descriptors produces a better model to predict the anti-*Mycobacterium tuberculosis* activities of these compounds. The QSAR model generated met the criteria for minimum recommended value of validation parameters for a generally acceptable QSAR model. The molecular docking analysis has shown that nearly all the 1,2,4-Triazole derivatives potentially inhibit Mtb CYP121. However, compounds 7, 8, 13, and 14 have higher bind score ranging from −10.03 to −11.02 kcal/mol. These four compounds were able to be docked deeply within the binding pocket region of the Mtb CYP121, forming a hydrogen bond and hydrophobic interactions with amino acid of the target. The QSAR model generated provides a valuable approach for ligand base design, while the molecular docking studies provide a valuable approach for structure base design. These two approaches will be of great help for pharmaceutical and medicinal chemists to design and synthesize new anti-*Mycobacterium tuberculosis* compounds.

## Figures and Tables

**Table 1:** Molecular structure of 1,2,4-Triazole derivatives and their activities.

S/N	Molecules	Experimental activity (pBA)
**1**	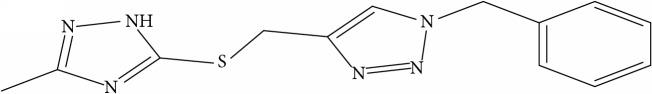	4.925

2^a^	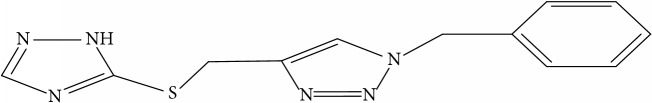	5.0345

**3**	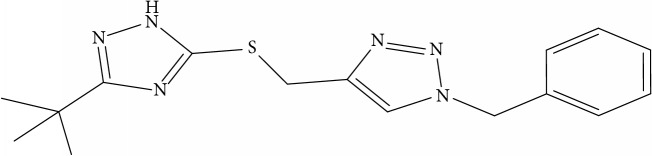	5.0064

**4**	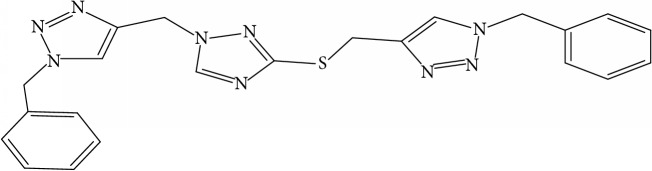	5.7386

5^a^	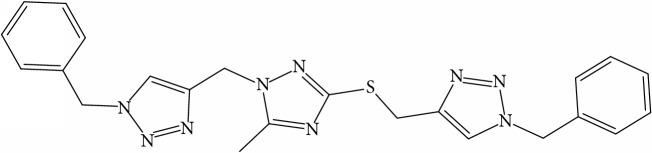	5.5994

6^a^	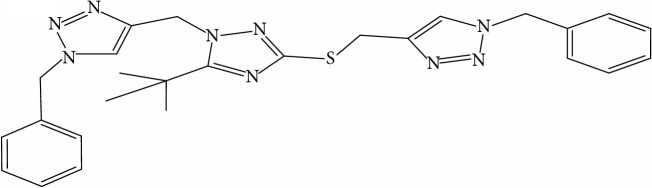	5.4543

**7**	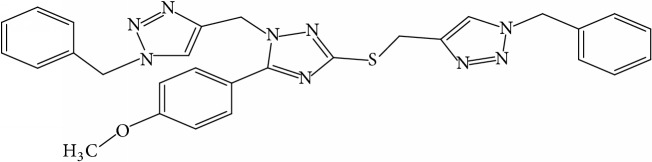	4.7441

**8**	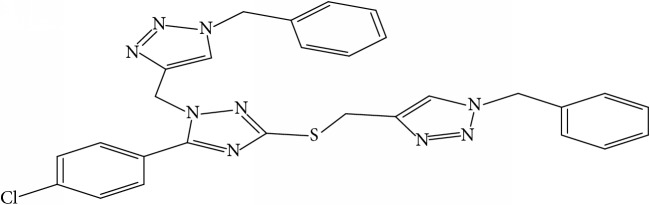	6.1674

9^a^	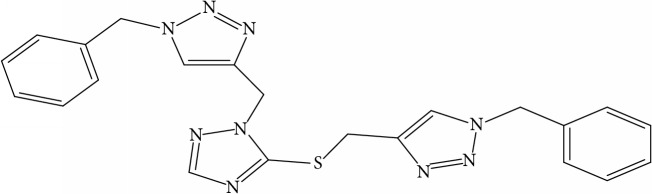	6.3456

**10**	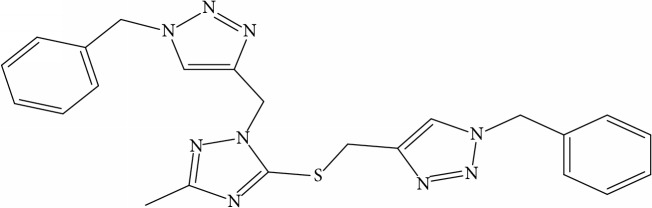	7.4134

**11**	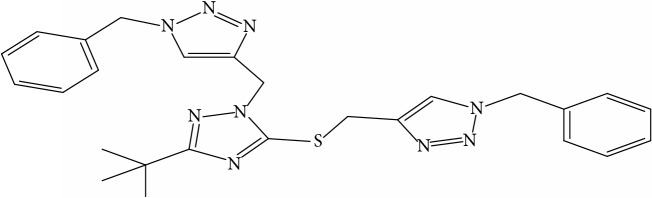	5.7441

**12**	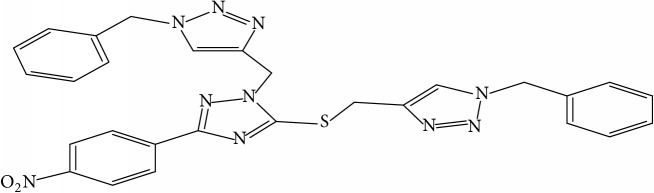	5.9258

13^a^	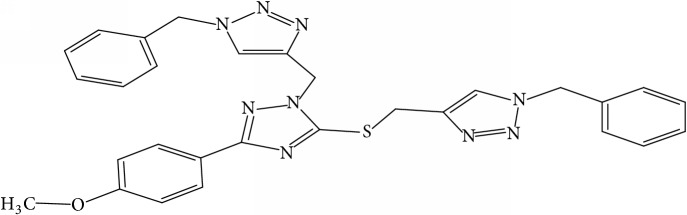	5.6754

**14**	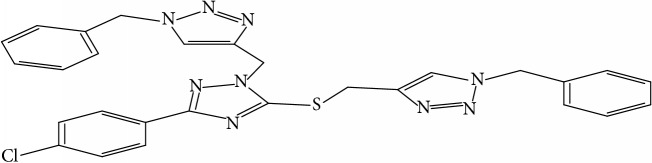	6.3793

**15**	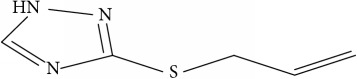	6.1667

16^a^	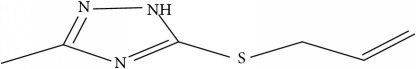	5.8765

**17**	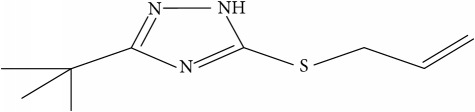	6.4171

**18**	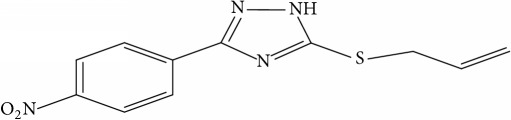	5.9413

**19**	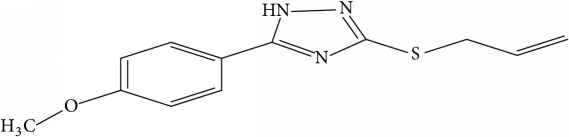	7.6397

**20**	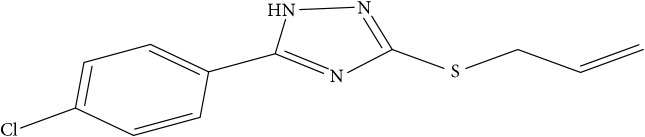	8.0899

**21**	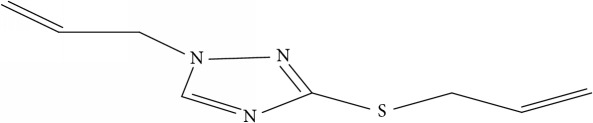	6.3981

**22**	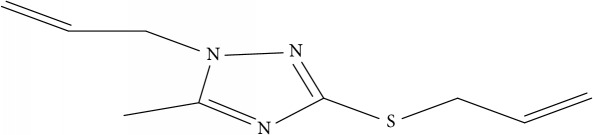	5.8131

**23**	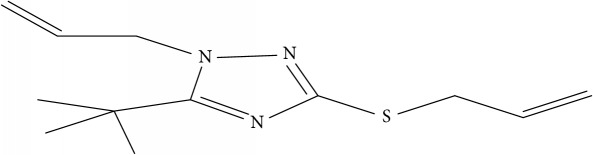	6.2878

**24**	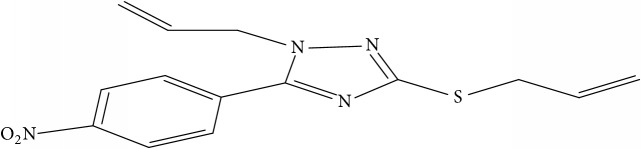	5.7268

**25**	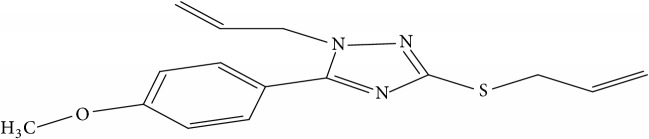	7.366

26^a^	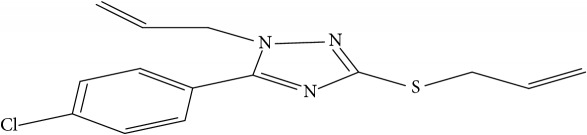	7.0123

**27**	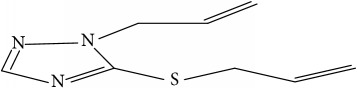	6.5267

**28**	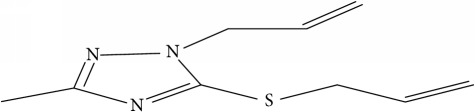	5.7405

29^a^	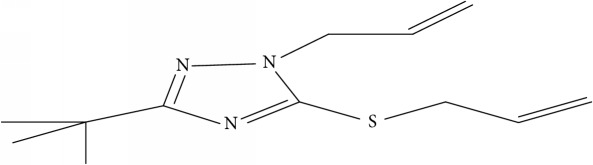	5.6533

30^a^	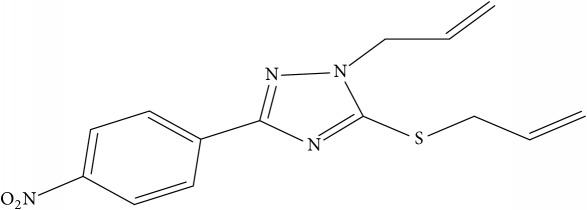	6.1923

**31**	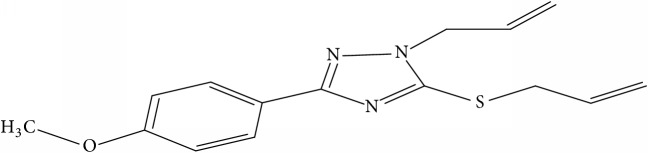	7.3233

**32**	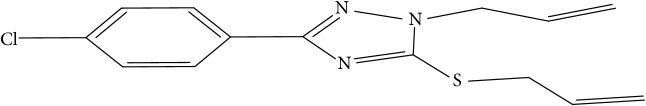	6.0097

**33**	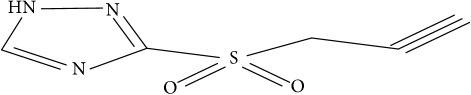	6.0928

**34**	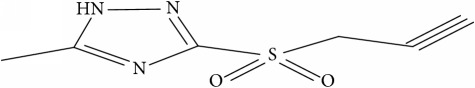	7.3279

**35**	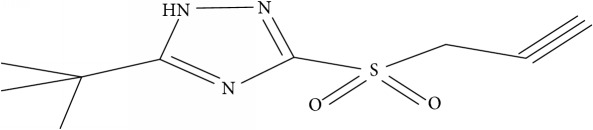	6.8568

36^a^	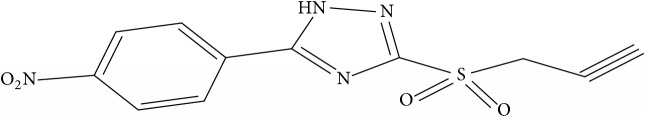	6.2234

**37**	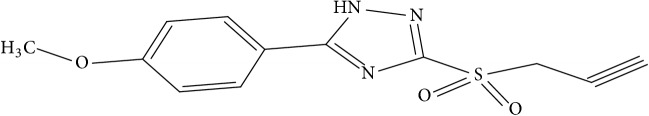	7.3079

**38**	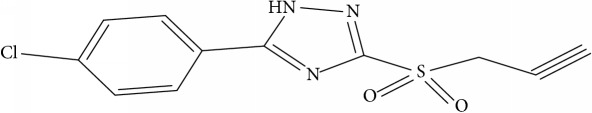	7.314

39^a^	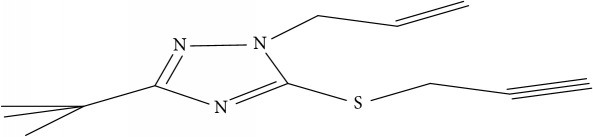	8.5854

**40**	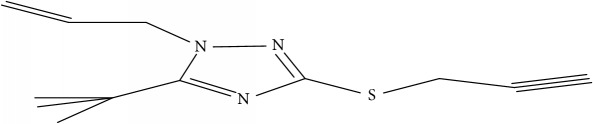	8.0615

**41**	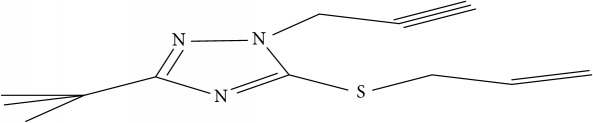	8.0615

42^a^	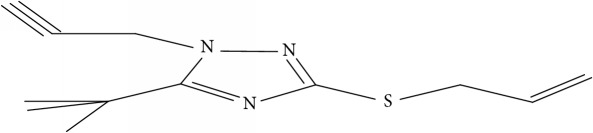	6.8494

**43**	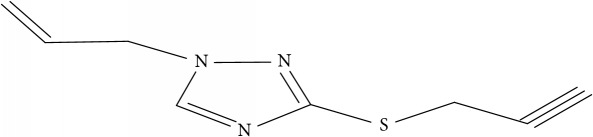	7.9432

44^a^	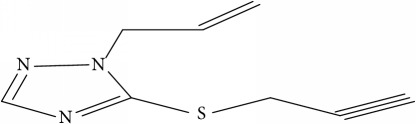	7.4535

**45**	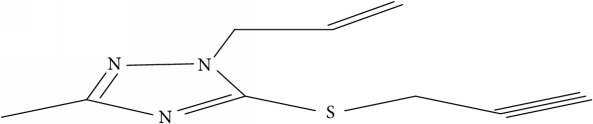	7.9759

**46**	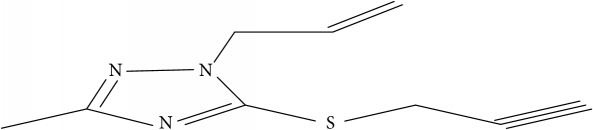	7.9759

**47**	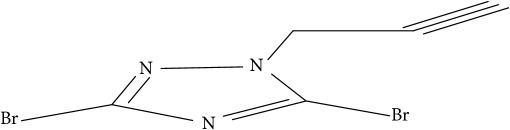	7.9294

48^a^	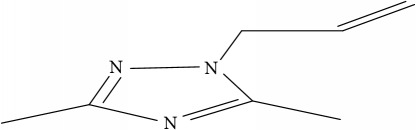	6.1213

**49**	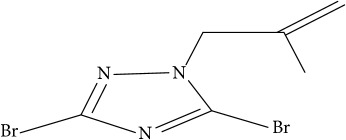	5.4406

50^**a**^	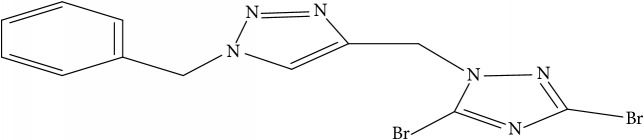	4.9074

Superscript **a** represents the test set.

**Table 2:** Minimum recommended value of validation parameters for a generally acceptable QSAR model.

Validation parameter	Name	Value
*R* ^2^	Coefficient of determination	≥0.6
*P*_(95%)_	Confidence interval at 95% confidence level	<0.05
*Q*_cv_^2^	Cross-validation coefficient	>0.5
*R* ^2^−*Q*_cv_^2^	Difference between *R*^2^ and *Q*_cv_^2^	≤0.3
*N*_ext. test set_	Minimum number of external test sets	≥5
*R* ^2^ test	Coefficient of determination for external test set	≥0.6
*cR*_*p*_^2^	Coefficient of determination for *Y*-randomization	>0.5

**Table 3:** Experimental, predicted, and residual values for 1,2,4-Triazole derivatives.

S/number (molecules)	Experimental activity (pBA)	Predicted activity (pBA)	Residual
**1**	4.925	4.8922	0.0328
**2**	5.0345	4.8716	0.1629
**3**	5.0064	5.0941	−0.0877
**4**	5.7386	5.8308	−0.0922
**5**	5.5994	5.5803	0.0191
**6**	5.4543	5.6969	−0.2426
**7**	4.7441	4.8047	−0.0606
**8**	6.1674	6.2999	−0.1325
**9**	6.3456	6.5053	−0.1597
**10**	7.4134	7.1548	0.2586
**11**	5.7441	6.0862	−0.3421
**12**	5.9258	5.6383	0.2875
**13**	5.6754	5.4834	0.192
**14**	6.3793	6.3443	0.035
**15**	6.1667	6.5432	−0.3765
**16**	5.8765	6.8765	−1.000
**17**	6.4171	6.1354	0.2817
**18**	5.9413	6.02517	−0.08387
**19**	7.6397	7.6055	0.0342
**20**	8.0899	7.8436	0.2463
**21**	6.3981	6.2094	0.1887
**22**	5.8131	6.4308	−0.6177
**23**	6.2878	6.30457	−0.01677
**24**	5.7268	5.9933	−0.2665
**25**	7.366	7.5444	−0.1784
**26**	7.0123	6.8471	0.1652
**27**	6.5267	5.9850	0.5417
**28**	5.7405	6.0962	−0.3557
**29**	5.6533	6.4796	−0.8263
**30**	6.1923	6.0426	0.1497
**31**	7.3233	6.5095	0.8138
**32**	6.0097	6.3151	−0.3054
**33**	6.0928	5.9501	0.1427
**34**	7.3279	7.3990	−0.0711
**35**	6.8568	6.8761	−0.0193
**36**	6.2234	8.6487	−2.4253
**37**	7.3079	7.2405	0.0674
**38**	7.314	7.5050	−0.191
**39**	8.5854	5.6969	2.8885
**40**	8.0615	8.1009	−0.0394
**41**	8.0615	7.8073	0.2542
**42**	6.8494	7.6746	−0.8252
**43**	7.9432	7.9352	0.008
**44**	7.4535	7.6946	−0.2411
**45**	7.9759	7.8569	0.119
**46**	7.9759	8.2103	−0.2344
**47**	7.9294	7.9408	−0.0114
**48**	6.1213	5.9165	0.2048
**49**	5.4406	5.2695	0.1711
**50**	4.9074	4.8495	0.0579

**Table 4:** Validation of the genetic function approximation from Materials Studio.

S/number		Equation
**1**	Friedman LOF	0.40847300
**2**	*R*-squared	0.92023900
**3**	Adjusted *R*-squared	0.91017400
**4**	Cross-validated *R*-squared (*Q*_cv_^2^)	0.89538600
**5**	Significant regression	Yes
**6**	Significance-of-regression *F*-value	58.41835200
**7**	Critical SOR *F*-value (95%)	2.45854700
**8**	Replicate points	0
**9**	Computed experimental error	0.00000000
**10**	Lack-of-fit points	28
**11**	Min expt. error for nonsignificant LOF (95%)	0.24688800

**Table 5:** Descriptive statistics of the inhibition data.

Statistical parameters	Activity	
	Training set	Test set
Number of sample points	35	15
Range	3.3458	3.678
Maximum	8.0899	8.2854
Minimum	4.7441	4.9074
Mean	6.622234	6.498873
Median	6.3981	6.1213
Variance	0.924712	0.866467
Standard deviation	0.96162	0.93084
Mean absolute deviation	0.871588	0.703515
Skewness	−8.48*E* − 04	0.87066
Kurtosis	−1.24682	0.153415

**Table 6:** List of some descriptors used in the QSAR optimization model.

S/number	Descriptors symbols	Name of descriptor(s)	Class
**1**	AATS7s	Average Moreau-Broto Autocorrelation-lag 7/weighted by I-state	2D
**2**	nHBint3	Count of E-state descriptors of strength for potential hydrogen bonds of path length 3	2D
**3**	minHCsatu	Minimum atom-type H E-state: H on C sp3 bonded to unsaturated C	2D
**4**	TDB9e	3D topological distance based autocorrelation-lag 9/weighted by Sanderson electronegativities	3D
**5**	RDF90i	Radial distribution function-090/weighted by relative first ionization potential	3D
**6**	RDF110s	Radial distribution function-110/weighted by relative I-state	3D

**Table 7:** Pearson's correlation matrix and statistics for descriptor used in the QSAR optimization model.

	Intercorrelation						Statistics	
Descriptors	AATS7s	nHBint3	minHCsatu	TDB9e	RDF90i	RDF110s	*t*-Stat	VIF
AATS7s	1						−3.9153	1.8931
nHBint3	−0.29824	1					11.6469	1.2779
minHCsatu	0.196097	0.269067	1				10.0386	3.6622
TDB9e	0.446768	−0.19131	−0.14868	1			5.66824	1.3493
RDF90i	0.097382	−0.13902	−0.39183	0.144839	1		9.45783	3.0968
RDF110s	0.116862	−0.25217	−0.66819	0.208747	0.227911	1	−5.5848	3.0275

**Table 8:** Specification of entered descriptors in genetic algorithm-multiple regression model.

Descriptors	Standard regression coefficient (*b*_*j*_)	Mean effect (ME)	*p* value (confidence interval)
AATS7s	−0.2769	−0.31421	0.000527
nHBint3	0.67675	0.153246	3*E* − 12
minHCsatu	0.987436	0.58264	8.84*E* − 11
TDB9e	0.338438	0.351968	4.48*E* − 06
RDF90i	1.097495	0.34097	3.25*E* − 10
RDF110s	−0.49948	−0.11461	5.62*E* − 06

**Table 9:** *Y*- Randomization parameters test.

Model	*R*	*R* ^2^	*Q* ^2^
Original	0.962302	0.926026	0.895386
Random 1	0.387394	0.150074	−0.28301
Random 2	0.534646	0.285847	−0.15518
Random 3	0.357333	0.127687	−0.43633
Random 4	0.509588	0.25968	−0.08884
Random 5	0.231807	0.053735	−0.60188
Random 6	0.140884	0.019848	−0.61556
Random 7	0.513288	0.263465	−0.11043
Random 8	0.548099	0.300412	−0.062
Random 9	0.36673	0.134491	−0.25601
Random 10	0.505524	0.255554	−0.12398

Random models parameters			
Average *r*	0.409529		
Average *r*^2^	0.185079		
Average *Q*^2^	−0.27332		
c*R*_*p*_^2^	0.837983		

**Table 10:** Binding affinity of 1,2,4-Triazole derivatives with *M. tuberculosis* target (Mtb CYP121).

Ligand	Target	Binding affinity (BA) Kcal/mol
**1**	Mtb CYP121	−7.3
**2**	Mtb CYP121	−7.8
**3**	Mtb CYP121	−8.5
**4**	Mtb CYP121	−9.1
**5**	Mtb CYP121	−9.6
**6**	Mtb CYP121	−9.8
**7**	Mtb CYP121	−10.3
**8**	Mtb CYP121	−14.6
**9**	Mtb CYP121	−9.6
**10**	Mtb CYP121	−9.6
**12**	Mtb CYP121	−9.2
**13**	Mtb CYP121	−11.2
**14**	Mtb CYP121	−11.2
**15**	Mtb CYP121	−5.1
**11**	Mtb CYP121	−9.9
**16**	Mtb CYP121	−5.3
**17**	Mtb CYP121	−6.1
**18**	Mtb CYP121	−7.9
**19**	Mtb CYP121	−7
**20**	Mtb CYP121	−7.8
**21**	Mtb CYP121	−5.5
**22**	Mtb CYP121	−5.7
**23**	Mtb CYP121	−5.5
**24**	Mtb CYP121	−6.9
**25**	Mtb CYP121	−6.6
**26**	Mtb CYP121	−6.7
**27**	Mtb CYP121	−5.4
**28**	Mtb CYP121	−5.1
**29**	Mtb CYP121	−5.4
**30**	Mtb CYP121	−7.5
**31**	Mtb CYP121	−7.3
**32**	Mtb CYP121	−6.6
**33**	Mtb CYP121	−5.6
**34**	Mtb CYP121	−6
**35**	Mtb CYP121	−6.3
**36**	Mtb CYP121	−7.8
**37**	Mtb CYP121	−7.8
**38**	Mtb CYP121	−8.4
**39**	Mtb CYP121	−5.7
**40**	Mtb CYP121	−6.3
**41**	Mtb CYP121	−6.3
**42**	Mtb CYP121	−5.9
**43**	Mtb CYP121	−5.6
**44**	Mtb CYP121	−5.5
**45**	Mtb CYP121	−6.2
**46**	Mtb CYP121	−5.7
**47**	Mtb CYP121	−5.9
**48**	Mtb CYP121	−5.7
**49**	Mtb CYP121	−5.2
**50**	Mtb CYP121	−7.8

**Table 11:** Binding affinity, hydrogen bond, and hydrophobic bond of ligands 7, 8, 13, and 14 with *M. tuberculosis* target (Mtb CYP121).

Ligand	Binding affinity (BA) Kcal/mol	Target	Hydrogen bond		Hydrophobic interaction
			Amino acid	Bond length (Å)	Amino acid
**7**	−10.3	Mtb CYP121	GLN385	2.16131	VAL83, PRO285, VAL78, VAL78, LA167
**8**	−14.6	Mtb CYP121	ALA337 HIS343 ALA233	2.82894 2.34089 2.47314	PHE280, ALA233, CYS345, MET86, ALA233, PRO346
**13**	−11.2	Mtb CYP121	ASN74 GLN385	2.34218 3.0328	VAL78, ALA233, PRO285, ALA233, PRO346 ALA167
**14**	−11.2	Mtb CYP121	ASN74 GLN385	2.36479 3.03627	LEU164, VAL228, VAL78, ALA233,PRO285 ALA233, PRO346, ALA167, ALA233
